# The influence of contextual factors on healthcare quality improvement initiatives: what works, for whom and in what setting? Protocol for a realist review

**DOI:** 10.1186/s13643-017-0566-8

**Published:** 2017-08-23

**Authors:** Emma Coles, Mary Wells, Margaret Maxwell, Fiona M. Harris, Julie Anderson, Nicola M. Gray, Gill Milner, Stephen MacGillivray

**Affiliations:** 1 Nursing Midwifery & Allied Health Professions Research Unit (NMAHP-RU), University of Stirling, Scion House, University of Stirling Innovation Park, Stirling, FK9 4NF UK; 2 Scottish Improvement Science Collaborating Centre (SISCC), School of Nursing and Health Sciences, University of Dundee, 11 Airlie Place, Dundee, DD1 4HJ UK

**Keywords:** Realist review, Realist synthesis, Context, Quality improvement, Health improvement, Implementation, Healthcare, Evidence-based practice, Knowledge translation

## Abstract

**Background:**

Context shapes the effectiveness of knowledge implementation and influences health improvement. Successful healthcare quality improvement (QI) initiatives frequently fail to transfer to different settings, with local contextual factors often cited as the cause. Understanding and overcoming contextual barriers is therefore crucial to implementing effective improvement; yet context is still poorly understood. There is a paucity of information on the mechanisms underlying *how* and *why* QI projects succeed or fail in given settings. A realist review of empirical studies of healthcare QI initiatives will be undertaken to examine the influence and impact of contextual factors on quality improvement in healthcare settings and explore whether QI initiatives can work in all contexts.

**Methods:**

The review will explore which contextual factors are important, and how, why, when and for whom they are important, within varied settings. The dynamic nature of context and change over time will be explored by examining which aspects of context impact at key points in the improvement trajectory. The review will also consider the influence of context on improvement outcomes (provider- and patient-level), spread and sustainability. The review process will follow five iterative steps: (1) clarify scope, (2) search for evidence, (3) appraise primary studies and extract data, (4) synthesise evidence and draw conclusions and (5) disseminate findings. The reviewers will consult with experts and stakeholders in the early stages to focus the review and develop a programme theory consisting of explanatory ‘context–mechanism–outcome’ configurations. Searches for primary evidence will be conducted iteratively. Data will be extracted and tested against the programme theory. A review advisory group will oversee the review process. Review findings will follow RAMESES guidelines and will be disseminated via a report, presentations and peer-reviewed publications.

**Discussion:**

The review will update and consolidate evidence on the contextual conditions for effective improvement and distil new knowledge to inform the design and development of context-sensitive QI initiatives. This review ties in with the study of improvement programmes as vehicles of change and the development of an evidence base around healthcare improvement by addressing whether QI initiatives can work in all contexts.

**Systematic review registration:**

PROSPERO CRD42017062135

## Background

### Quality improvement in healthcare

Evidence-based healthcare is the translation of evidence derived from valid and reliable research and evaluation to improve healthcare practice and patient outcomes [1]. Quality improvement (QI) is the systematic application of specific methodologies to make improvements in how services are configured and delivered. The Health Foundation, in defining quality improvement, highlights ‘the combination of a ‘change’ (improvement) and a ‘method’ (an approach with appropriate tools), while paying attention to the context, in order to achieve better outcomes [2]. Healthcare improvement is defined by Batalden and Davidoff as combined efforts to make changes leading to ‘better patient outcomes (health), better system performance (care) and better professional development’ [3]. However, variable, inconsistent results and the often limited impact of improvement initiatives [4] suggest that translating evidence into practice and implementing healthcare innovations is not straightforward. One of the key problems in the implementation of evidence-based interventions using QI methodologies is that what works in one setting does not always readily transfer to other settings, suggesting that many improvements are context-dependent [5]. This evidence-into-practice or implementation gap is often attributed to the ‘problem’ of context, i.e. the variations in a wide range of contextual conditions and factors across different settings that can impact on the improvement process, influencing the implementation, effectiveness, spread and sustainability of QI initiatives [4, 6–9].

### What is context?

At its most simplistic, context can be defined as ‘all factors that are not part of a quality improvement intervention itself’ [8], i.e. ‘the set of characteristics and circumstances or unique factors that surround a particular implementation effort’ [6]. Similarly, Kaplan et al. [10] view context as distinct from the QI implementation process. The SQUIRE 2.0 publication guidelines, recently updated to include context as a fundamental item in improvement reporting, view context as the ‘key features of the environment in which the work is immersed and which are interpreted as meaningful to the success, failure, and unexpected consequences of the intervention(s), as well as the relationship of these to stakeholders (e.g. the improvement team, clinicians, patients)’ [11]. However, locating context as largely outside the QI process and intervention implementation may be insufficient. Context is not merely the backdrop to implementation: it interacts, influences, modifies, facilitates or constrains the intervention and its effectiveness [12, 13]. Indeed, contextual factors are frequently conceptualised within implementation research literature as barriers and facilitators to effective implementation [14]. The importance of individual contextual elements can be dependent on the type of intervention being implemented and its infancy or maturity, the stage of implementation or the level at which it is targeted, and, in the case of multi-component interventions, specific components [9].

The theories underpinning an intervention and its implementation also contribute to the context [15]. Accordingly, intervention, implementation and context are highly intertwined, with all three interacting with and influencing each other [16]. Hence, it can be difficult to separate an intervention from its context, not least because the boundaries are relatively arbitrary [8] so there are inevitably interactions over time between the two [5, 17]; hence, the implementation of interventions within dynamic contexts means that boundaries become blurred [18]. May also highlights the dynamic nature of context, defining context as ‘complex adaptive systems that form the dynamic environment(s) in which implementation processes are situated’ [19]; additionally, contextual ‘confounders’ that act as a barrier in one setting may facilitate implementation in another [20].

Traditional QI approaches, generally focusing on efficacy, often paid little attention to context, with contextual factors ignored or poorly reported in implementation reporting [21, 22]. However, interest in context has increased in recent years: there is now a growing recognition of the importance of context, its multi-faceted and complex nature and its influence on knowledge implementation and healthcare improvement efforts [23–25]. It is accepted that the ‘fit’ between intervention and the different levels of context is crucial to success [14], although questions remain as to whether improvement interventions should be adapted to fit their context, or attempts should be made to modify local contexts in order to successfully implement change [23]. Whichever course of action is chosen, a complete understanding of the role and influence of context is vital; elucidating and overcoming contextual barriers at the local level is essential to implementing improvement strategies that are not only effective but also sustainable and transferable. Nonetheless, until relatively recently, there has been little guidance as to which aspects of context are most important and at what stage of the improvement cycle, and whether they can be modified to enhance the impact of evidence-based QI interventions. Furthermore, across the literature definitions of context vary, and often overlap with similar concepts, such as setting and environment, suggesting that the conceptualisation of context is not yet fully developed [16]. Being able to define and assess context is vital for the transferability of QI interventions across multiple settings [21]. In addition, given that we need to recognise the interactions between contextual influences and understand their relative importance, it may be appropriate to take a more theoretically driven and explanatory approach [26] to advance our understanding of context and the role it plays in the success or otherwise of improvement initiatives [23].

### Context in improvement: an emerging field

The increased interest in context in recent years has resulted in an emergent number of implementation frameworks and models, many including hierarchical checklists of factors to enable assessment of context influences at different system levels by clinicians and QI teams and to aid organisations with the design and implementation of their improvement efforts [13, 27–30] although not all are based on empirical data [6]. Most frameworks primarily focus on implementation, with context one of a range of aspects considered [22]. An overview of key published frameworks and their features is summarised in Table 1.

**Table 1:** Published healthcare improvement frameworks

Framework	Description and focus	Features
Context and Implementation of Complex Interventions (CICI) Pfadenhauer et al. 2016 [13]	Facilitates conceptualisation and assessment of context in implementation of complex interventions. Focuses on understanding and/or explaining what influences implementation outcomes.	Three integrated dimensions—context, implementation and setting—which interact with one another and with the intervention dimension. Context comprises seven domains: geographical, epidemiological, socio-cultural, socio-economic, ethical, legal, and political.
The Model for Understanding Success in Quality (MUSIQ) Kaplan et al. 2012 [29]	Attempts to embed context in QI efforts. Targeted at microsystem and organisational efforts.	Identifies 25 key contextual factors at different levels of the healthcare system that are likely to influence the success of quality improvement efforts. Five domains: microsystem, QI team, QI support and capacity, organisation, and external environment.
Consolidated Framework for Implementation Research (CFIR) Damschroder et al. 2009 [6]	Implementation framework comprising of constructs from 19 published implementation theories. For studying and planning implementation of health services research findings into practice. Provides a basis for studying context influences.	Integrates implementation, setting and context. Five domains: intervention, outer setting, inner setting, individuals, and process. Outer setting, inner setting and the characteristics of individuals are considered aspects of context.
Context Assessment Index (CAI) McCormack et al. 2009 [28]	Validated instrument for QI context assessment in implementing evidence-based practice. Allows clinicians to assess and understand the context in which they work and the effect this has on using evidence in practice.	37-item instrument. Assesses three elements of context: culture, leadership and evaluation.
Promoting Action on Research Implementation in Health Services (PARiHS) Kitson et al. 2008 [27]	Implementation framework targeted at implementation of evidence and innovations. Used to explain and/or predict success of evidence into practice implementation.	Presents successful research implementation as a function of the interactions between three key domains: evidence, context and facilitation.

Alongside these frameworks, there is a growing body of literature on context in QI. Existing reviews of improvement studies and reviews of reviews in this field have focused on identifying, describing and categorising contextual factors that influence QI implementation and effectiveness [7, 17, 31], thus providing a valuable contribution to the debate, although Portela et al. [32] suggest the need to move beyond traditional systematic reviews towards synthesis methods that allow for greater conceptual development.

Despite the fact that new methods of identifying and addressing context are emerging, and understanding about the influence of context in QI is increasing, evidence suggests that the science of assessing or measuring contextual factors is still ‘immature’ [26]. Context is still often absent from empirical QI research [33] and frequently fails to be acknowledged, described or taken into account during implementation; for example, few studies assess the impact of context on implementation, and often, QI reports provide only basic information on contextual features [8]. This may be partly due to choice of study design; many methodologies do not facilitate exploration of *how* interventions work, unlike for example, theory-driven and realist evaluation, which seeks to understand the interactions between intervention, variations in context and underlying mechanisms of change [32]. Although traditional experimental and quasi-experimental methods are useful in order to learn whether or not QI initiatives cause change, quantitative assessments of effectiveness are not always able to articulate the ‘how’ and ‘why’, or the realist questions, about improvement: what works, for whom and under what circumstances [32, 34].

### What will this review add?

The variability across the literature on context and its associated disparate body of evidence suggests that further work is required in this area. Therefore, the question remains: in terms of improvement methodologies and implementation, which characteristics of context matter, and how, why, when and for whom do they matter? Furthermore, how can this knowledge interface with current and future health improvement work? Existing reviews focusing on effectiveness have not tended to address the underlying causal mechanisms by which context influences evidence-based practices and why QI initiatives do or do not work in given contexts [5], although notably, the call for an increased understanding of how such mechanisms interact with context is not new [35]. Yet these mechanisms of change are still not well understood, thus hindering replicability, with many measures of context lacking theoretical underpinning [7, 32, 36]. Given that there are gaps in the literature, a more explanatory approach (exploring *how* and *why*) is required to unpick the complex interactions between context, QI intervention and outcomes. This realist review aims to bridge these gaps by providing a context-sensitive evidence synthesis focusing on the influence of contextual factors on QI implementation and transferability.

This review ties in with the study of improvement programmes as vehicles of change and the development of an evidence base around healthcare improvement by addressing the question of whether improvement programmes can work in all contexts. Studying the role of context in impacting on the implementation of ‘improvement methodologies’ (as opposed to interventions) is a key part of this learning. This suggests the need to collate information on the settings and circumstances in which improvement methodologies are effective, capturing the multi-level and multi-factorial nature of contextual influences, and to identify the successful components that potentially are generalizable across different programmes. Dissemination of such knowledge would support a more context-sensitive approach to the design and implementation of improvement initiatives, incorporating the influence of local context into the design of new interventions and the evaluation of existing programmes. Not only will this be of importance to academic researchers but ultimately will be of practical value to QI practitioners, on an individual, team and organisational level, in real-world settings.

By testing and refining existing theories of context, this realist review will address Bate’s concern that ‘we do not need a new model or framework to study the role of context in quality improvement; rather, we need to test and synthesise existing ones’ [37]. In updating the evidence base to incorporate the more recent advances in the context and QI literature—a rapidly developing field—this review also offers potential to take forward one of the conclusions of the recent Health Foundation review of the evidence for context which states: ‘The evidence base for intervening to modify contextual factors in order to positively impact on the outcomes of quality improvement interventions is currently very weak.’ [17]. This is a particularly important issue given that many QI approaches currently do not address contextual factors ‘up-front’ [38], instead making adaptations during later stages of the improvement cycle; considering context in advance would facilitate the scale and spread of improvement.

### Why a realist review of context?

Realist reviews, building iteratively on theoretical and empirical literature, are able to accommodate and synthesise a diversity of evidence types [39]; indeed, realist inquiry allows reviewers to look beyond randomised controlled trials (RCTs) as the primary source of review evidence [40]. RCTs do not tend to answer the ‘how’ and ‘why’ questions [1] that are central to realist methodology, and crucially, experimental designs exclude contextual influences [8]. Moreover, concerns have been expressed about the quality of QI randomised trials [41]. There is a need to look beyond experimental studies: previous reviews and overviews of or related to context in implementation have included few qualitative studies, tending to focus on quantitative primary studies [17, 26], yet qualitative methodologies are more able to address the how and why questions surrounding QI effectiveness and implementation in given contexts. In sum, given the potentially broad scope of the topic area (QI in healthcare) and the need to include a broader range of evidence types than can be accommodated within a traditional systematic review framework, a realist approach [39], with its iterative nature and application of theory-driven literature searching, was deemed most appropriate.

Realist inquiry focuses on taking an explanatory approach with an emphasis on understanding causation. It is based on the assumption that all programmes and interventions have underlying theories—whether implicit or explicit—about what might cause change and that these theories may be modified as evidence emerges [15, 39]. Thus, it is a flexible, theory-driven approach that can accommodate the complexity of interventions and the real-world relationships between social and contextual factors and human behavioural responses that can influence the success or failure of a programme and contribute to a range of diverse outcomes and effects, whether intended or not. Hence, the logic of realist explanation can be used to better understand the implementation and effective delivery of complex health interventions and improvement programmes. Furthermore, the realist context–mechanism–outcome (CMO) model is ideally suited to exploring the context sensitivity of QI interventions and the contextual factors surrounding their implementation [21], in that it facilitates more nuanced explanations than traditional systematic reviews can offer, by exploring how and why each contextual factor is important, and increasing understanding of how and why people respond to interventions (the mechanisms of change). Indeed, context as a key realist tenet will allow inferences about context to be drawn from studies where contextual issues may not be explicitly articulated. However, issues around the clarity of the concepts of context, mechanism and outcome, and the challenges of distinguishing between them and assessing their causal relationships are acknowledged [42–45].

Despite the relative newness of the method, realist inquiry has been utilised in recent reviews in the field of evidence-based healthcare improvement [46–51]. The review team are also able to draw on their own prior experience of realist review and realist evaluation [52–54].

### Aims of the review

The overarching research question to be addressed is the following: how and why does context influence healthcare QI initiatives? The primary aims of this realist review are to (i) identify the contextual factors that influence the implementation, effectiveness, sustainability and transferability of quality improvement initiatives in healthcare; (ii) provide a theoretical explanation of how, why, when and for whom these contextual factors are important and (iii) transform the wealth of data in this area into a cohesive evidence base of ‘what works, for whom and in what circumstances’, in terms of implementation in given contexts. A further, secondary aim concerns the interface between research, policy and practice. After completion of the review, findings will be translated into evidence-based, practical knowledge and recommendations that can be shared with and applied by policymakers and practitioners.

### Scope of the review

For the purposes of this review, quality improvement in healthcare is defined as ‘better patient experience and outcomes achieved through changing provider behaviour and organisation through using a systematic change method and strategies’ [55], broadly encompassing quality, safety, effectiveness, patient-centeredness, timeliness, cost, efficiency and equity [11]. A broad definition of healthcare is used, incorporating adult and paediatric physical and mental health, and inclusive of health behaviour change, in order to ensure transferability of review findings to wider health and social care settings. English language primary or empirical studies (e.g. realist evaluations, process evaluations, feasibility studies) reporting on the design and/or implementation of QI initiatives (inclusive of individual interventions, wider programmes, large-scale transformative change and QI collaborative efforts) and evidence-based healthcare improvement within healthcare settings in developed industrialised countries will be considered for inclusion. Searching will be restricted to studies published in the previous 10 years; the evidence base is likely to be more fragmented before this point given that not much more than a decade ago, QI was considered a relatively new and developing field for health service research [56]. Additionally, contextual issues are less likely to be explicitly acknowledged or reported in older studies.

### Collaboration

At the beginning of the review process, a multi-disciplinary review advisory group will be set up to support the review team, oversee progress, and help develop consensus by providing a forum for monitoring, discussion and the sharing of feedback at all stages. This group will initially consist of the review team and members of the Scottish Improvement Science Collaborating Centre (SISCC), a multi-sectoral network of researchers, educators and practitioners across academia and health and social care. The composition of the group may change or expand during the course of the review; other identified experts and stakeholders may be invited to collaborate with the research team as the review progresses. The group will contribute to all aspects of the review from theory development to interpretation of findings.

## Methods

The review will follow the fundamentals of realist inquiry, a theory-driven interpretive approach that seeks to explain the root causes of programme outcomes and diverse patterns of impacts and effects, by evaluating evidence from a range of sources [39]. The aim of realist inquiry is explanation-building…‘to articulate underlying programme theories and then to interrogate the existing evidence to find out whether and where these theories are pertinent and productive. Primary research is examined for its contribution to the developing theory’ [39]. Accordingly, the realist approach centres on the development and refinement of theory, in order to provide plausible and evidenced explanations, in descriptive narrative form, as to why and how interventions may or may not work, in multiple contexts and with multiple actors [57, 58]. The principal output of a realist review, or realist synthesis—the terms are used synonymously [59]—is an evidence-based, iteratively tested and refined ‘programme theory’: an articulation of how and why a programme or family of programmes is thought to cause its desired outcomes within various contextual configurations.

In realist thinking, programmes are theories [39]. The hypotheses (ideas, beliefs and intended outcomes) that lie behind each intervention or family of interventions, outlining how they are meant to work, are identified, tested and refined through the course of the realist review, with the aim of examining the extent to which these hypotheses are met in practice. Programme theories are expressed using the interlinked concepts of ‘context’ (C), ‘mechanism’ (M) and ‘outcome’ (O). Programmes attempt to alter context in some way (for example, by the provision of resources), thus affecting existing mechanisms or introducing new ones, to produce desired outcomes. Mechanisms are the responses triggered by the context, which cause outcomes, whether intended or unintended. Mechanisms will produce different outcomes in different contexts. The way in which causation occurs for any given outcome is expressed by the formula *C* + *M* = *O*; a programme theory may consist of multiple CMO configurations that explain the context, mechanism and outcome relationships and the pattern of outcomes. By recognising the importance of external and environmental influences, realist inquiry can be used to unpack complex programmes and interventions and explore the reasons underlying how and why they do or do not work in given contexts [15].

The review will be conducted following the five key stages of Pawson’s realist review template [39]: (1) clarify scope, (2) search for evidence, (3) appraise primary studies and extract data, (4) synthesise evidence and draw conclusions and (5) disseminate findings. The stages are presented in a linear fashion, but in practice, unlike traditional systematic reviews, they are conducted iteratively with some overlap to be expected. Accordingly, refinements to the developing programme theory are made throughout the review process based on emerging findings. Further, realist review methodology is distinctive in that there are at least two search phases. In stage 1, relatively unstructured ‘broad brush’ scoping searches are undertaken to provide an overview of the area of study and, in realist terms, identify and map underlying programme theories. This serves to narrow the scope of the review and contribute to the development of a theory-based review framework in the form of a realist programme theory. Stage 2 of the review process involves conducting purposive, theory-driven searches for additional evidence to test and refine the programme theory.

### Clarifying the scope of the review: exploratory searching, mapping and theory development

As the area of interest is potentially very wide-ranging, a priority at the first stage will be to refine and narrow the research question via a broad-based scoping exercise. It is at this point that key themes and concepts are defined as the focus of the review begins to take shape and the reviewers begin to develop a theoretical understanding of the research topic. Following Pawson’s methodology [39], relatively unstructured exploratory internet-based searches will be carried out, guided by a limited number of combinations of search terms related to the topic of context in healthcare-related quality improvement. These searches will be purposefully broad in order to locate a varied range of evidence (including reviews, theories, models and frameworks) which can provide an overview of the topic area. The scoping searches will be augmented by specific, highly focused searches for key evidence as well as existing evidence already known to the review team and associates. This initial immersion in the literature not only allows the reviewers to gain a deeper understanding of the research problem but primarily serves to facilitate the identification of, in realist terms, the underlying ‘programme theories’—in this case, explanations of how QI methodologies and interventions interact with their context and how and why they are supposed to work in order to produce their desired outcomes. Therefore, the exploratory search stage forms part of an initial scoping exercise to (a) find key literature in the field and (b) identify and map the range of existing programme theories in order to create a theoretical framework for subsequent stages of the review. The aim is to uncover the programme theories within the literature; these may be in the form of assumptions about how and why a QI initiative is intended to work (i.e. the causal mechanisms), dominant themes, explicit or implicit references to theory, descriptions of the ways in which change is anticipated to occur or theoretical descriptions of the linkage between programme activities and outcomes.

As the scoping exercise progresses and findings begin to emerge, the reviewers will develop an outcome-focused realist programme theory, based on the identified existing programme theories and structured around explanatory context–mechanism–outcome (CMO) configurations, consisting of propositions about the ways in which contextual factors are anticipated to influence improvement initiatives in practice.

In keeping with realist methodology, key stakeholders and experts, including QI practitioners, will be consulted throughout this first stage in order to provide a range of additional perspectives and an ‘expert framing’ of the issues that can contribute to the developing programme theory. Consultation will take place in person or by email. Theory development will also be informed by meetings of the review advisory group. The resulting theoretical, explanatory model and its component contexts, mechanisms and outcomes of interest will be used as the framework for the succeeding stages of the review.

### Search for primary evidence

Systematic searches for primary studies that are relevant to the programme theory will then be carried out. This second search phase will involve seeking additional relevant evidence (e.g. process evaluations and qualitative research) primarily to test and refine the programme theory. The search process and appraisal of evidence will be theoretically driven, purposive and conducted iteratively. Searching will be progressively extended and refined, based on emerging findings, as the review evolves, until saturation is reached. The aim of realist review is theoretical saturation as opposed to fully comprehensive coverage of the topic area. Selection of sources will be based on relevance to aspects of the programme theory.

A combination of search strategies will be utilised. Internet search engine and electronic database searching will be carried using keywords based on the programme theories identified in the exploratory search. This will be supplemented by a ‘cited by’ article search and a search of citations included in the reference lists of included papers. Sources of grey literature, including unpublished reports, will also be investigated, as well the websites of relevant organisations, such as The Health Foundation and the Institute for Healthcare Improvement. The reviewers will make use of snowballing techniques and consultation with experts and stakeholders. Given that a wide range of documents may contain data that can contribute to a realist review, multiple types of evidence will be included. Based on the data retrieved and emerging findings, the direction of the review may shift or expand in scope.

Search results from electronic databases and other sources will be imported into reference management software (Endnote) and duplicates removed.

### Appraise evidence and extract data

The realist appraisal and extraction process differs from a traditional systematic review, as inclusion and exclusion criteria are based on the programme theory and what the literature is able to contribute to it. In a realist review, the units of analysis are not the interventions themselves per se but the theories underpinning the interventions. Accordingly, evidence will be selected for inclusion based on its relevance to the programme theory; the screening process will be based on an assessment of ‘fit’ to the research question, with the aim of identifying evidence that can empirically test, revise or refine the programme theory (or elements of it). ‘Bespoke’ data extraction forms [58] will be developed, incorporating key themes and questions based on the emerging findings and the programme theory developed in stage 1. Unlike data extraction forms used in traditional reviews, these will be used primarily to gather information on contextual factors, mechanisms and outcomes, along with additional data on QI implementation, intervention resources and so on, thus providing a template to interrogate the evidence. Outcomes of interest will include both implementation (process) outcomes and effectiveness (clinical) outcomes. The key test for evidence in a realist review is an assessment of its relevance (whether it can contribute to theory building and/or testing) and rigour (whether the methods used to generate the evidence are credible and trustworthy) [59]. The included evidence will be appraised for relevance and rigour using a ‘fitness for purpose’ approach, following the criteria outlined in the RAMESES (realist and meta-narrative evidence synthesis group) Quality Standards for Realist Synthesis [60], while bearing in mind that even methodologically weak studies that would be otherwise excluded by a traditional systematic review may contain potentially valuable ‘nuggets’ of understanding relevant to the review [39, 40]. Extracted data will be put into evidence tables and organised into themes. Data extraction will be carried out by at least two reviewers; interpretation of the data will be guided by the judgement and reflexivity of the review team. Any differences will be resolved through discussion with the review advisory group.

Again, in contrast to the traditional review process, during this stage, the project team will revisit and if necessary revise the focus of the review based on emerging findings. This may require further refinement of the inclusion/exclusion criteria and further purposive searches in response to any revisions of the programme theory, followed by the integration of any additional evidence.

### Analysis and synthesis of evidence: test/refine theory and draw conclusions

The key analytic process in a realist review involves iterative testing and refinement of theoretically based explanations (i.e. the programme theory) using empirical findings in data sources [59]. The goal of the fourth stage is thus to test and refine the initial programme theory by drawing comparison with the primary evidence and exploring and analysing the relationships between contexts, mechanisms and outcomes. Relevant passages of included documents will be annotated and coded to identify contexts, mechanisms, outcomes and CMO configurations. The reviewers will compare and contrast the evidence, looking for recurring patterns of CMOs across the data that are able to support, contradict or inform the programme theory. This is an iterative process, guided by the research question and primary aims of the review. Completion of the realist synthesis will allow the reviewers to modify or refine the identified CMO configurations, and use these to explain (a) how and why QI initiatives cause change and generate outcomes within particular contexts and (b) which contextual factors matter, and how, when and for whom they matter, in terms of their influence on the QI process. It is at this point that overall conclusions are drawn and a set of tentative recommendations produced. This penultimate stage will enable the production of a final synthesis integrating review evidence with programme theory, and culminating in a revised programme theory, refined in light of the evidence and reflecting the review findings. Presented as a narrative, the findings will be written up according to the publication standards outlined by the realist and meta-narrative evidence synthesis (RAMESES) group [60] and will follow the format set out by the RAMESES standards.

### Dissemination of findings

Findings will be translated into evidence-based, practical knowledge and recommendations that can be shared with and applied by policymakers, and QI researchers and practitioners. They will be disseminated in the form of a final report, presentations to stakeholders and peer-reviewed publications.

## Discussion

This study will use a realist review approach to synthesise the available evidence and to enable a greater understanding of ‘what works, for whom, in what circumstances, when, how and why’, in terms of the influence of contextual factors on quality improvement initiatives in healthcare settings, thus providing a new insight into the ‘problem of context’ in QI. The use of a realist approach will allow the review to describe and explain how and why QI initiatives work (or fail to work) in different contexts by exploring the underlying programme theories and the interactions between contextual factors, mechanisms of change and outcomes.

Synthesising current knowledge on evidence of context (research-based evidence of contextual factors affecting implementation) within quality improvement and making this knowledge available and accessible to stakeholders will facilitate the design and development of evidence-based, context-sensitive improvement activities that can be planned and delivered in a way that takes this latest evidence into account, mitigating for known contextual barriers and enhancing facilitators in advance wherever possible and incorporating local knowledge to enhance implementation and improvement strategies and improve transferability. This approach will ensure that current knowledge of contextual barriers can be taken into account in designing or planning for implementation and that ‘common’ or evidence-based contextual problems are not repeated from the outset but are mitigated for in planning, including tailoring of local plans.

## Abbreviations

CMO: Context–mechanism–outcome
PRISMA-P: Preferred Reporting Items for Systematic Reviews and Meta-Analyses Protocols
QI: Quality improvement
RAMESES: Realist and meta-narrative evidence syntheses—evolving standards
